# A mathematical model of adult subventricular neurogenesis

**DOI:** 10.1098/rsif.2012.0193

**Published:** 2012-05-09

**Authors:** J. M. A. Ashbourn, J. J. Miller, V. Reumers, V. Baekelandt, L. Geris

**Affiliations:** 1 Department of Engineering Science, University of Oxford, Parks Road, Oxford OX1 3PJ, UK; 2 St Hugh's College, St Margaret's Road, Oxford OX2 6LE, UK; 3 Laboratory for Neurobiology and Gene therapy, Katholieke Universiteit Leuven, Kapucijnenvoer 33i (7001), 3000 Leuven, Belgium; 4 Biomechanics Research Unit, Université de Liège, Chemin des Chevreuils 1 B52/3, 4000 Liège, Belgium; 5 Prometheus, Division of Skeletal Tissue Engineering, Katholieke Universiteit Leuven, Herestraat 49, 3000 Leuven, Belgium

**Keywords:** neurogenesis, mathematical model, sloppiness, sensitivity analysis, chemotaxis

## Abstract

Neurogenesis has been the subject of active research in recent years and many authors have explored the phenomenology of the process, its regulation and its purported purpose. Recent developments in bioluminescent imaging (BLI) allow direct *in vivo* imaging of neurogenesis, and in order to interpret the experimental results, mathematical models are necessary. This study proposes such a mathematical model that describes adult mammalian neurogenesis occurring in the subventricular zone and the subsequent migration of cells through the rostral migratory stream to the olfactory bulb (OB). This model assumes that a single chemoattractant is responsible for cell migration, secreted both by the OB and in an endocrine fashion by the cells involved in neurogenesis. The solutions to the system of partial differential equations are compared with the physiological rodent process, as previously documented in the literature and quantified through the use of BLI, and a parameter space is described, the corresponding solution to which matches that of the rodent model. A sensitivity analysis shows that this parameter space is stable to perturbation and furthermore that the system as a whole is sloppy. A large number of parameter sets are stochastically generated, and it is found that parameter spaces corresponding to physiologically plausible solutions generally obey constraints similar to the conditions reported *in vivo*. This further corroborates the model and its underlying assumptions based on the current understanding of the investigated phenomenon. Concomitantly, this leaves room for further quantitative predictions pertinent to the design of future proposed experiments.

## 1. Introduction

Neurogenesis has been the subject of active research in recent years and many authors have explored the phenomenology of the process, its regulation and its purported purpose in both the subventricular zone (SVZ) and the dentate gyrus of the hippocampus. In the adult SVZ, type-B astrocytes that are present close to endothelial cells act as slowly dividing neural stem cells, capable of generating a progeny of type-A neuroblast precursors [1] through a rapidly proliferating type-C cell intermediate. Type-A neuroblasts then migrate in a unique fashion to the olfactory bulb (OB) along a path known as the rostral migratory stream (RMS), which is large (of the order of millimetres) in comparison with the size of each individual cell (of the order of microns) [2]. These neuronal precursors migrate in chains along the RMS, which is ensheathed by astrocytic processes and outlined by blood vessels [3–5]. After migrating through the RMS, these type-A neuroblasts arrive at the centre of the OB and then move radially outwards. They further specify into either OB granule cells or OB periglomerular cells, and become fully developed mature neurons that stain with NeuN but are otherwise indistinguishable from OB cells [1]. Thus, to a first approximation, the stem cells differentiate linearly in a cascade throughout their complex spatial journey.

During migration, type-A cells continue to divide and initiate neuronal maturation [6], but their rate of proliferation is drastically reduced and the cell cycle time is lengthened [7]. The migration is predominantly towards the OB, but many parts of the individual paths can point in other directions. In answer to the question of what guides migration, it is often theorized that differentially expressed migration factors might be responsible for steering migration, and a very large number of factors have been shown to influence the process: brain-derived neurotrophic factor [8]; growth factors such as endocrine growth factor [9], nerve growth factor [10] and fibroblast growth factor-2 [11]; polysialylated neural cell adhesion molecules, [12] and many other substances have been shown to have a mixed effect upon the entire neurogenic process [13]. It therefore appears probable that an attractant factor in the OB might direct migrating type-A neuroblasts towards the bulb. However, cells migrate successfully even when the OB is surgically separated from the rest of the brain [14], although the magnitude of migration is greatly reduced. Insertion of an explant culture of OB cells subsequently ameliorates the process, but insertion of any other piece of brain tissue does not, nor do many known chemoattractants [15]. This implies that attractant factors are both present within the OB and secreted from the cells in either an endocrine or paracrine fashion, or are present within the wider environment; the exact mechanism remains currently unknown.

Recent developments in bioluminescent imaging (BLI) have allowed direct *in vivo* imaging of neurogenesis within rodents over a prolonged time period and the measurement of a number of tagged cells temporally throughout the process and spatially throughout the brain [8]. Properties such as cell migration speeds, proliferation, specification and apoptosis rates undoubtedly have a significant influence upon the results of such experiments and would prove troublesome to quantify in rodents. Furthermore, the specific mechanism and chemical factors responsible for the regulation of the process are at present largely unknown as outlined earlier. We therefore propose a simplified mathematical model, the results from which will, we hope, guide further experimentation. Theoretical and computational modelling of neurogenesis is not new. It has been used to study processes during development [16] as well as in the postnatal and adult brain [17,18]. Aimone *et al*. [16] review a suite of neurogenesis models, classifying them into ‘abstract’, anatomy independent models and ‘biological’ models that encompass specific details of the anatomical location. The latter category has been used to study processes in the hippocampus [19–23] and the OB [17]. What these models have in common though is their focus on the functional effect of neurogenesis. In this study, we do not aim to consider all the details of the functional effect of the processes we model; instead the focus here is on providing a tool to interpret the experimental measurements. The agreement between the results of this model and the physiological process lends credence to the assumptions upon which the model relies, most notably that migratory neuronal chemoattractants are secreted both at a constant rate by both the environment and in an endocrine/paracrine manner by the migrating cells.

## 2. Material and methods

### 2.1. Model and assumptions

**Figure 1.**
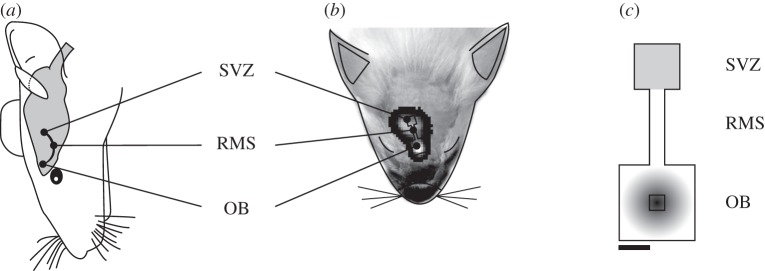
(*a*) Illustration of the approximate physiological location of the migratory process. (*b*) Location of the computational domain with inset a typical BLI. (*c*) The three interconnected boxes that form the computational domain; the initial concentrations of the chemoattractant factor *f*_A_ and *n*_B_ cell density are shown in grey within the olfactory bulb (OB) and subventricular zone (SVZ), respectively. The central black box indicates the region considered to be the centre of the OB, where in this simplifying model all type-A neuroblasts which arrive either undergo apoptosis or specify into adult neurons. RMS, rostral migratory stream. Scale bar shows unit length in the model, corresponding to 1.4 mm if dimensionalized.

Spatially, the migration of cells through the RMS is a complex three-dimensional process [1,13,24], but to date quantifying BLI only has occurred in two-dimensional axial slices. We therefore consider that modelling the process in two dimensions at greatly increased computational and mathematical ease is an acceptable simplification. We assume that the computational domain can take the form of two interconnected boxes, the larger corresponding to the anatomically larger OB and the smaller to the much smaller SVZ, as illustrated in figure 1. Given the fact that we are modelling neurogenesis in the adult brain, domain growth can be neglected, which further simplifies the mathematical framework [25].

**Figure 2.**
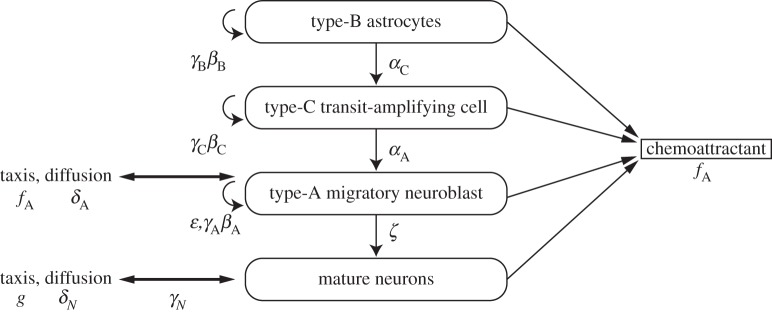
A schematic overview of the model and its terms, illustrating the linear differentiation cascade of the neuroblasts and associated terms. *γ* and *ε* are apoptosis rates; *β* are proliferation rates; *α* and *ζ* are differentiation rates; *δ* are diffusion constants; *f* and *g* are chemoattractants.

Figure 2 shows a schematic overview of the model presented in this study. It is generally acknowledged that type-B cells in the SVZ specify into type-C transit-amplifying cells, which then become the migratory type-A neuroblasts. These type-A migratory neuroblasts do not change their morphology further before either becoming adult neurons or undergoing apoptosis [13]. Although a small proportion (1.6%) of type-A cells seem to be able to become type-E endothelial cells [26] while in the SVZ itself, we do not model the density of the structural type-E cells but rather include this small loss of type-A cells within their apoptotic term. Because this is constant spatially within the model, this is tantamount to assuming that the rate of growth of supporting endothelial cells is constant spatially throughout the brain. Hence, we assume that every cell specifies in a linear way (B → C → A in the SVZ) and we denote the rate of specification by *α_i_n_i_*, *i* = A, B, C with *n_i_* = *n_i_*(**r**, t); *i* = A, B, C being the cell density of type-A, B, C cells, **r** the two-dimensional position vector and *t* the non-dimensionalized time. We allow type-A, -B and -C cells to undergo mitosis and apoptosis at different rates, and we assume that these rates are not a function of position for each cell, apart from the type-A migratory neuroblasts. As type-A cells have been reported to proliferate much more slowly outside the SVZ than within it and a large proportion of the new cells arriving in the OB die [27], we take the type-A cells to have two different rates of mitosis with logistic growth constants *β*_A*i*_ within the SVZ, and *β*_Ao_ outside it as well as two different rates of apoptosis, (*γ*_A_ + *ε*)*n*_A_ within the central region of the OB and *γ*_A_*n*_A_ elsewhere in the domain.

We take type-B and type-C cells to be fixed in space and we let type-A cells move under the influence of a single chemoattractant factor, *f*_A_(**r**,*t*). This factor is assumed to be diffusible, taken up by the type-A cells at a rate *κ*_D_*f*_A_*n*_A_, able to undergo some natural decay *λf*_A_, produced by the other species of cell present at a rate *κ_i_n_i_* and produced by the OB environment at a rate
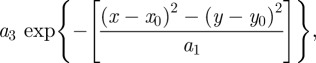
where the location of the centre of the OB is (*x*_0_*,y*_0_) and *a*_1_ and *a*_3_ are positive constants. As a significantly simplifying step, we ignore the complex radial development and migration of type-A migratory neuroblasts into granule cells or periglomerular cells [28], and we assume that type-A cells either specify into adult neurons (with cell density *n_N_*) or die [27], once they reach a region surrounding the centre of the OB. For computational ease, the central region is a square of side *b* and we define *ε*(**r**) and *ζ*(**r**) as two related functions that are non-zero only within it; *ε* is the increased rate of type-A cell apoptosis as described earlier, and *ζ* = *με* is the rate of mature neuron formation within the OB, with *μ* being a positive constant. We further assume that the mature neurons are diffusible, have a slow rate of apoptosis (*γ_N_n_N_*; *γ_N_* ≪ *γ_i≠N_*) and, as a model simplification, undergo taxis against a constant environmental factor *g* that is present in the form of a Gaussian to represent the radial migration found in the physical system. If we denote diffusion constants by *δ_i_* and taxis coefficients by *η_i_*, this leads to the following set of equations:2.1
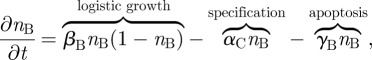
2.2
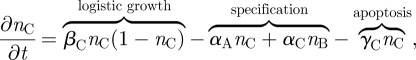


2.3
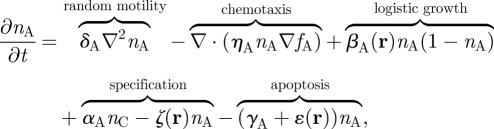


2.4
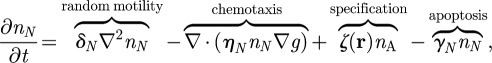
2.5
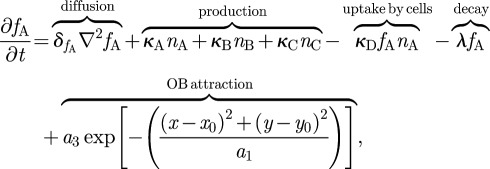


2.6
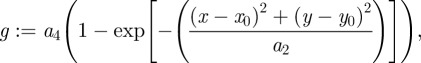
2.7
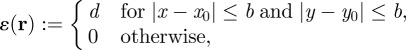
2.8
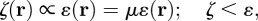
2.9
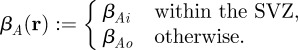


The system of partial differential equations (PDEs) defined earlier was closed with appropriate boundary (zero-flux) and initial conditions. According to the BLI experiment, the initial concentrations of mature neurons, type-C and type-A cells were set at zero, with type-B cells and the factor *f*_A_ having an initial two-dimensional Gaussian distribution in the centre of the SVZ and the OB, respectively. The model was run for 300 days according to the experimental set-up [8].

### 2.2. Implementation

The variables in this system of differential equations represent concentrations, either of cells or of chemical factors. It is therefore vital that the numerical solutions to such a system are never negative, in particular because allowing negativity for any brief period of time may introduce unphysical oscillations and greatly affect the stability of the system. In addition, the method of solution used must ensure the conservation of mass and impose zero-flux conditions at the boundary of the computational domain to represent the constraints both of this model and of anatomy. The algorithm used to solve this taxis–diffusion–reaction system was that presented by Gerisch & Chaplain [29]. This implementation uses the method of lines (MOLs) to separate the spatial and temporal discretization of differentials over the computational grid of 15 600 spatial points across the domain. The diffusion and reaction terms in equations (2.1)–(2.5) were discretized using the standard second-order central difference approximation and pointwise evaluation method, respectively, at the central point of each grid cell, both of which were found to be sufficient in terms of accuracy. As reported previously [29,30], the discretization of the taxis terms required the application of upwinding techniques with the non-linear van Leer limiter function to guarantee accurate, positive solutions for the system of ordinary differential equations (MOL-ODEs). For the time domain, the efficient numerical integration scheme ROWMAP was used, a ROW-code of order 4 with Krylov techniques for large stiff ODEs [31], which has an automated choice of step-size to ensure that the local error caused in each iteration remains below a user-prescribed tolerance (in this case 10^−6^), while keeping the computational cost as low as possible.

### 2.3. Model parameters

Parameter values were inferred (in non-dimensional form) from the BLI dataset. We stochastically generated values for each of the parameters over a range in magnitude (10^−6^–10^6^) using a Mersenne twister algorithm [32] for the pseudo-random number generator, with the majority of the solutions investigated using generated values for each parameter that lie in the region (0,10). The reason for this choice of space was pragmatic: as the whole system is non-dimensional, scaling the magnitude of every parameter essentially redefines the coordinate axes. By (manual) comparison between solutions from different parameter sets (stochastic or otherwise), a guided search for physiological solutions was initiated, which ceased when a parameter space could be described that did not contain unphysical solutions [33]. Generating purely random parameters within the interval (0,1) resulted in physiological solutions twice in 180 runs (1%); guided stochastic searches in narrower domains increased this to 30–40%. Searches for new parameter values were ceased when 200 random samples of a space defined by a ±10 per cent perturbation to a physiologically appealing parameter space (described later) resulted in entirely physiological solutions. Computational time for parameter sets leading to physiological solutions was around 1800 s on a single core of an Intel Xeon Harpertown 5500 processor, whereas solutions leading to steep variations, large gradients, pattern formation or quantities close to zero were computationally more expensive. Of the system of equations (2.1)–(2.5), it is apparent that equations (2.1) and (2.2) have algebraic steady-state solutions of the form2.10
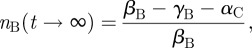
2.11
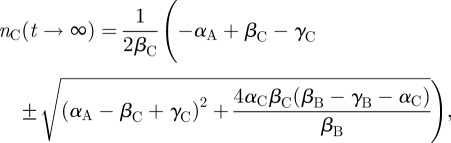
where the unphysical solution corresponding to *n*_B_ = 0 is ignored. We therefore constrained the parameter space by requiring equations (2.10) and (2.11) to be greater than zero in order to avoid trivial solutions. To avoid solutions where neurons randomly diffused through the RMS rather than through undergoing taxis, we required *η_i_* > *δ_i_*. Other biological taxis models [30] frequently have the diffusion term three orders of magnitude smaller than the taxis one; hence this constraint is not particularly stringent in context. To check that these conditions were sufficient for physical solutions, a small number of spaces that did not satisfy the positive steady-state conditions but with larger initial conditions were explored, in order to allow the possibility for taxis within a long period of non-equilibrium behaviour; unphysical oscillations were observed as the only result.

### 2.4. Parameter and model sensitivity analysis

The sensitivity of the solution to small perturbations in the parameter set was investigated in a straightforward manner by considering the space defined by subjecting each parameter value to a ±10 per cent perturbation, stochastically sampling this space 200 times and then solving the resulting systems. This showed that spaces yielding physiological solutions with plausible taxis times (i.e. non-zero and approximately within an order of magnitude of the experimental data) are not unique. As is common in biological models, one has to consider the system as a whole and observe correlations and correspondence with experimental data; little can be gained by trying to accurately determine the values of a small range of individual parameters. This problem is well documented, and similar to Gutenkunst *et al.* [34], we defined a generalized parameter-space least-squares metric *ζ*^2^ that quantifies how the solutions change throughout parameter space relative to one particular set of parameter coordinates θ* as2.12
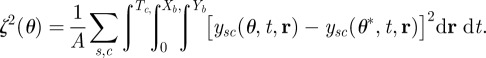


Here *t* represents the time ordinate, *θ* represents the parameter space coordinates, *X_b_* and *Y_b_* are the maximum extent of the computational domain in the *x-* and *y-*directions, *T*_c_ the time up to which the simulation was run and *A* = 2*·T*_c_*N*_s_ is a normalization factor with *N*_s_ being the number of species within the model and the sum extending over all species present. The value of *ζ*^2^ was computed for every physical (non-oscillatory) solution obtained via fast trapezoidal numerical integration (trapezoidal integration performed successively across each dimension). Because of the fact that solving the PDE system is computationally intensive and most traditional ways of creating a representation of *ζ*^2^ would require a large number of solutions, we elected to use a non-linear interpolation method to find approximations of *ζ*^2^ straightforwardly; an interpolation method that does not require an evenly spaced grid of function value points was chosen to allow the calculation of *ζ*^2^ for all solutions obtained. In this way, the stochastic generation of parameter values represents a Monte-Carlo approach to finding *ζ*^2^. The ‘obtuse angle’ interpolation method was used [35] and found to provide an adequate approximation to the highly non-linear *ζ*^2^ function. The validity of this approach was checked by comparison with results obtained using other methods of non-linear interpolation, such as the use of radial basis functions, which displayed some evidence of fitting bias. Furthermore, in order to investigate the error in the approximation to *ζ*^2^, simulations were run with parameters randomly perturbed by ±10 per cent compared with θ*, i.e. within the whole range that the interpolation function was trained on. These parameters were noted, and the differences between interpolated and computed metrics evaluated. This indicates an average relative error of approximately 50 per cent (*m* = 0.49, *n* = 10). Close to a data point, relative error is significantly better.

As documented by Gutenkunst *et al.* [34], the Hessian matrix of all possible second-order derivatives of the function *ζ*^2^, i.e. the matrix
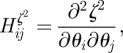
has eigenvectors that are the principal axes of the such ellipsoids and has eigenvalues that span many orders of magnitude. We obtained this matrix from the interpolated approximation to *ζ*^2^ via standard numerical high-order finite difference approximations (as detailed in [36]) with the use of suitable small step sizes. The value of having this matrix as a function of *θ* (rather than θ*) is that it could form a basis for a collective parameter fit in future studies [25].

### 2.5. Experiments

The experimental set-up considered in this study is the same as that presented in the study by Reumers *et al*. [8] where BLI was used to track *in vivo* stem cell migration. In summary, neural progenitor cells were transduced with lentiviral vectors encoding firefly luciferase that was injected into the SVZ of mice. BLI was used to detect and quantify the progeny of these transduced cells in the OB over a period of 45 weeks post-injection. On day 7 post-injection, a detectable BLI signal was present at the site of the injection and from week 4, a signal from the OB could also be detected. A full report of the experimental set-up and the results is given in [8].

## 3. Results

**Figure 3.**
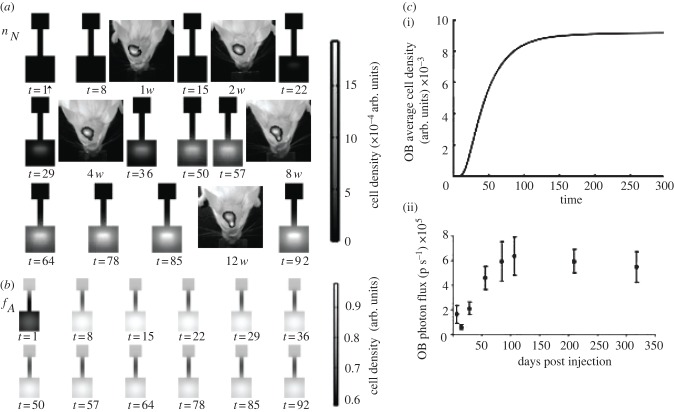
(*a*,*b*) The emergence of mature neurons in the system and the time evolution of the chemotactic factor *f*_A_ for the set of parameter values *β*_B_ = 7.567, *β*_C_ = 9.441, *β*_A*i*_ = 1.735, *β*_A*o*_ = 0.785, *α*_C_ = 2.182, *α*_A_ = 1.700, *γ*_B_ = 0.701, *γ*_C_ = 0.774, *γ*_A_ = 0.737, *γ_N_* = 0.079, *δ*_A_ = 0.037, *δ_N_* = 0.053, *δ_fA_* = 1.955, *η*_A_ = 0.134, *η_N_* = 9.136, *κ*_A_ = 6.045, *κ*_B_ = 1.276, *κ*_C_ = 2.292, *κ*_D_ = 9.284, *λ* = 0.422, *a*_1_ = 1.000, *a*_2_ = 0.500, *a*_4_ = 1.000, *a*_5_ = 0.461, *b* = 0.500, *d* = 1.921, *μ* = 0.213. Initial conditions were *n*_C_, *n*_A_, *n_N_* = 0; *n*_B_ and *f*_A_ initially obeyed a Gaussian distribution: *f*_A_ in the OB with a maximum magnitude of unity and a standard deviation of half of the OB's side; *n_N_* in the SVZ with a maximum magnitude of 100 and standard deviation of 5*/*6 × half of the side of the SVZ. (*c*)(i) A comparison between the OB cell density of the model (*n*_A_ + *n*_N_) and (ii) recorded *in vivo* bioluminescent photon fluxes of the OB reported in [8]. (p s^−1^); photons per second.

Solutions to this system broadly fell into two categories: those that were physiologically plausible and those that were patently not. Those that we considered plausible showed signs of a steady, controlled migration, the relatively quick equilibration of *n*_B_ and *n*_C_ to their steady-state values and a steady growth of mature neurons in the OB over time until a maximum value was reached. An example of such a set of solutions in comparison to the mouse BLI [8] is shown in figure 3. Taking the non-dimensional unit of time to be days, there is a good agreement between the model predictions and the experimental *in vivo* results, in terms of both the qualitative behaviour of the cells and the timing of the process.

As discussed in §2.2, a number of constraints were placed on the parameter space from which solutions were generated; we found that *δ_f_*_A_ ≫ *δ_a_*_,_*_n_*, which is indicative of molecular as opposed to cellular diffusion. Furthermore, we found that the logistic growth constants for all three types of cells within the SVZ were usually within the same order of magnitude. Spaces with migration-related parameter values that were representative of those that might be expected from the literature—*β*_C_ ≫ *β*_A_,*β*_B_—often resulted in solutions that were not unphysical, but where the taxis of type-A cells was small with respect to the effect of death terms. This either resulted in no neuronal maturation within the OB or in a quick transition to a steady-state constant flux of neurons through the OB, and not in the extended buildup over time that has been observed. Solutions deemed to be exhibiting unphysical behaviour showed either signs of exponentially growing oscillations or spontaneous symmetry breaking with the onset of divergent pattern formation. The electronic supplementary material, S1 contains an overview of all the constraints on the parameter space needed to generate physiological solutions, along with the parameter set used as a reference in this study.

**Figure 4.**
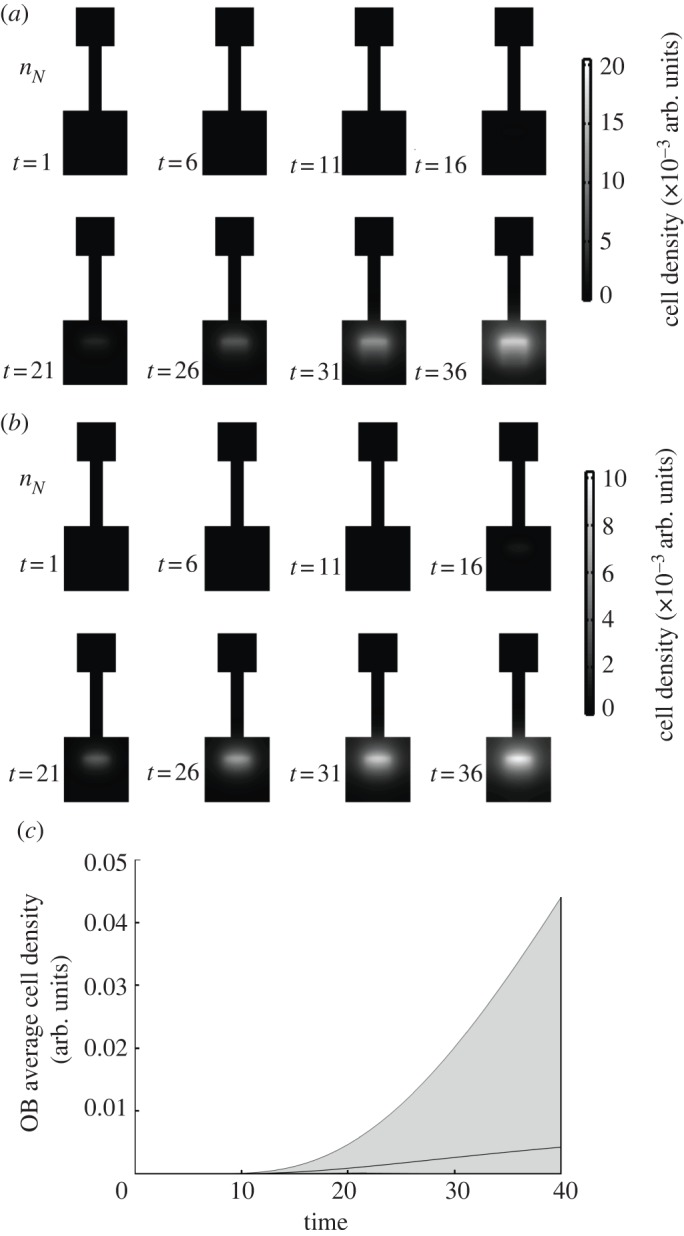
A direct comparison between the most and least neurogenic (*a* and *b*, respectively) solutions obtained through a ±10*%* perturbation to the parameter space illustrated in figure 3. While the cell density in the OB varies greatly in magnitude between the two spaces (upper and lower bounds on the shaded region in (*c*); unperturbed solution in black), the overall behaviour changes very little.

The sensitivity to small perturbations in the solution illustrated in figure 3 and of others that were similarly close to the observed behaviour *in vivo* was investigated straightforwardly by subjecting every parameter value to a random ±10 per cent perturbation and solving the resulting system. For the solution illustrated in figure 3, 200 such perturbed systems were generated and solved, and we observed no unphysical behaviour in any of these results. While the equilibrium densities of cells predictably changed, the overall qualitative properties of the solutions remained the same and even solutions with radically different equilibrium densities displayed similar behaviour, as illustrated in figure 4.

**Figure 5.**
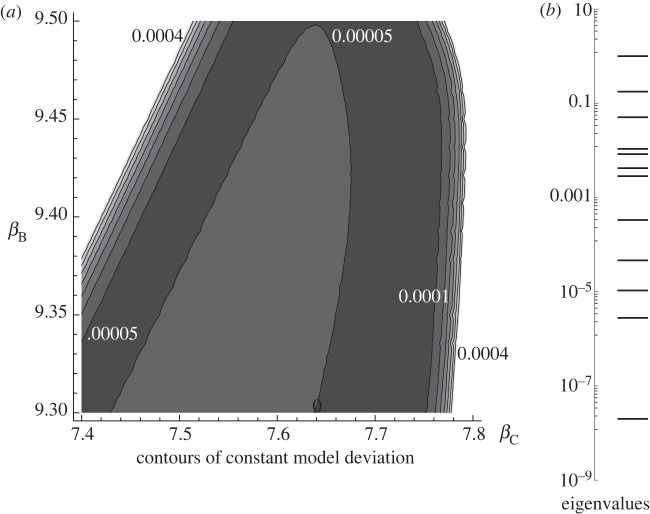
(*a*) Illustrative contours of constant model deviation as quantified by *ζ*^2^ (generalized parameter-space least-squares metric quantifying how solutions change throughout parameter space relative to one particular set of parameter coordinates); slices are through the *β*_B_–*β*_C_ plane (logistic growth constants for B- and C-type cells) with all other values being the same as those in figure 3. Contour values are labelled; the centre of the figure corresponds to a minimum. (*b*) The normalized absolute values of the eigenvalues of the Hessian matrix in the same parameter space location. These eigenvalues span nearly 10 orders of magnitude; a characteristic of sloppy systems [32].

In the sloppy parameter analysis, we observed multi-dimensional ellipsoids for the *ζ*^2^ function, as shown in figure 5*a*. This quantifies how the results of the model change as a function of its parameters, and therefore allows one to understand which parameters have the greatest effect upon the model outcome. Directions in parameter space, where a small change in parameter values results in a large change in *ζ*^2^, are referred to as ‘stiff axes’; those, where *ζ*^2^ deviates little, are known as ‘sloppy’. As illustrated in figure 5*b*, the magnitude of the eigenvalues of the Hessian matrix showed a substantial variation by decades. The eigenvalues span some 10 orders of magnitude when normalized by the value of the largest, indicating that the sloppiest axes of the ellipsoids illustrated are more than 10^5^ times as long as the stiffest. Furthermore, the values are spread out within this interval, thus indicating that there is no sharp distinction between ‘important’ and ‘unimportant’ parameter combinations and confirming the sloppy nature of the model under investigation [34]. This sloppiness is manifest in our failure to change the qualitative behaviour of the solutions by adjusting individual terms (e.g. decreasing migration time by increasing the magnitude of taxis terms *η*).

## 4. Discussion

In this study, we propose a mathematical model describing adult mammalian neurogenesis occurring in the SVZ and the subsequent migration of cells through the RMS to the OB. This model assumes that a single chemoattractant is responsible for cell migration, secreted both by the OB and in an endocrine fashion by the cells involved in neurogenesis. The solutions to the system of PDEs were compared with the physiological rodent process as observed by BLI [8] and a parameter space was described for which the corresponding solution matched that of the rodent model. A sensitivity analysis was carried out showing that the described parameter space is stable under perturbation and furthermore that the system as a whole is sloppy. A large number of parameter sets were stochastically generated, and it was found that parameter spaces corresponding to physiologically plausible solutions generally obey constraints similar to conditions reported *in vivo* in the literature.

We note that a steady decay from the maximum OB photon flux (which is proportional to the cell density) is observed in the BLIs, but not in our model; we propose that this is owing to cells within the rodent being tagged within the SVZ initially at one point in time, which will then eventually die [37]. This model tracks all neurons generated within the SVZ and therefore we expect to see no decline with time once a steady state has been reached. In addition, this model does not suffer from experimental problems such as stereotactic surgery scars, which have been seen around the site of the SVZ injection in the rodent images in figure 3*a*.

A number of simplifications have been made in the course of this study. A two-dimensional domain was used to model subventricular neurogenesis, which is a frequently performed simplification in many mathematical models of biological processes. Furthermore, given that the RMS is a predominantly planar system, we do no expect the results to fundamentally change when incorporating the third dimension. Another simplification lies in the numerical method used to calculate the parameter-space least-squares metric *ζ*^2^, i.e. through the use of a non-linear interpolation method. The highly non-linear nature of the metric could lead to larger errors away from the interpolation points which might influence the exact value of the derived Hessian. The representation of the migration used in this study is also a simplification. It is difficult to find or derive appropriate constraints for migration parameters from the literature. The majority of studies investigate the regulatory effect of a plethora of factors upon neurogenesis. The subsequently available constraints have either been derived from *in vitro* experiments, which can only study migratory behaviour on artificial surfaces, or from slices of the brain [38–40]. While the former might be far from the physiological environment, the latter could be incomplete when, for example, sources of chemoattraction are not present in the studied section of the brain. BLI now allows us to investigate this migratory behaviour *in vivo* for the first time.

Owing to the complexity of the underlying biology (involving not only migration with chemoattractants either binding or not to the extracellular matrix, but also cell differentiation, proliferation and death) it is not straightforward to derive quantitative information without the help of mathematical modelling. This study is a first step in this direction as it tries to incorporate the underlying biological mechanisms that influence the experimental output. Future research will focus on the incorporation of different migratory mechanisms. The model proposed in this study contains many ‘free’ parameters for which no values were available in the literature. Directly measuring them would be difficult for some parameters and impossible for others, especially in an *in vivo* environment. Collectively fitting the parameters to experimental data often yields large parameter uncertainties, as is the case for our study. However, it has been shown extensively in the literature that collective fitting in biological models could yield well-constrained predictions even when it left individual parameters very poorly constrained [34,41,42]. The results presented in [34] suggest that sloppy sensitivity spectra, as observed by the substantial variation in eigenvalue magnitudes in the model presented here, are universal in systems biology models. This prevalence of sloppiness highlights the power of collective fits and suggests that the focus of modelling efforts should be on predictions rather than on parameters.

## 5. Conclusion

We have shown how a relatively simple system of differential equations can accurately model the biological process of subventricular neurogenesis and that the individual parameter values of this system are of equal importance in determining the properties of its solutions. We have found a particular parameter space (which is probably not to be unique) that is stable to perturbation and that yields results that match others in the literature. We believe that this model could be extended and reapplied, once more accurate information on the various parameters is available, in order to make quantitative predictions of neurogenic behaviour. Furthermore, the agreement between this model and the BLI data implies that at least one chemoattractant factor is secreted in an endocrine/paracrine manner and is responsible for directing migration, as it is produced by all types of neural stem cells present. Whether this is physiologically the case remains to be confirmed experimentally.
